# Practical use of dabigatran etexilate for stroke prevention in atrial fibrillation

**DOI:** 10.1111/ijcp.12147

**Published:** 2013-04-05

**Authors:** K Huber, S J Connolly, A Kher, F Christory, G-A Dan, R Hatala, R G Kiss, B Meier, B Merkely, B Pieske, T Potpara, J Stępińska, N Vene Klun, D Vinereanu, P Widimský

**Affiliations:** 1 3rd Department of Medicine (Cardiology and Emergency Medicine), Wilhelminen HospitalVienna, Austria; 2 Division of Cardiology, Hamilton Health Sciences, McMaster UniversityHamilton, Ontario, Canada; 3 Consultant in Hemostasis and ThrombosisParis, France; 4 Medical Education Global SolutionsParis, France; 5 Cardiology Department, “Carol Davila” University of MedicineBucharest, Romania; 6 National Cardiovascular InstituteBratislava, Slovakia; 7 Department of Cardiology, Military Hospital, State Health CenterBudapest, Hungary; 8 Department of Cardiology, Bern University HospitalBern, Switzerland; 9 Heart Center, Semmelweis UniversityBudapest, Hungary; 10 Division of Cardiology, Department of Internal Medicine, Medical University of GrazGraz, Austria; 11 Faculty of Medicine, University of BelgradeBelgrade, Serbia; 12 Intensive Cardiac Therapy Clinic, Institute of CardiologyWarsaw, Poland; 13 Department of Angiology, University Clinical Center LjubljanaLjubljana, Slovenia; 14 Carol Davila University of Medicine and Pharmacy, 2nd Department of CardiologyBucharest, Romania; 15 Cardiocenter, Third Faculty of Medicine, Charles UniversityPrague, Czech Republic

## Abstract

Atrial fibrillation (AF) is associated with an increased risk of thromboembolism, and is the most prevalent factor for cardioembolic stroke. Vitamin K antagonists (VKAs) have been the standard of care for stroke prevention in patients with AF since the early 1990s. They are very effective for the prevention of cardioembolic stroke, but are limited by factors such as drug–drug interactions, food interactions, slow onset and offset of action, haemorrhage and need for routine anticoagulation monitoring to maintain a therapeutic international normalised ratio (INR). Multiple new oral anticoagulants have been developed as potential replacements for VKAs for stroke prevention in AF. Most are small synthetic molecules that target thrombin (e.g. dabigatran etexilate) or factor Xa (e.g. rivaroxaban, apixaban, edoxaban, betrixaban, YM150). These drugs have predictable pharmacokinetics that allow fixed dosing without routine laboratory monitoring. Dabigatran etexilate, the first of these new oral anticoagulants to be approved by the United States Food and Drug Administration and the European Medicines Agency for stroke prevention in patients with non-valvular AF, represents an effective and safe alternative to VKAs. Under the auspices of the Regional Anticoagulation Working Group, a multidisciplinary group of experts in thrombosis and haemostasis from Central and Eastern Europe, an expert panel with expertise in AF convened to discuss practical, clinically important issues related to the long-term use of dabigatran for stroke prevention in non-valvular AF. The practical information reviewed in this article will help clinicians make appropriate use of this new therapeutic option in daily clinical practice.

**What's known**

Three new oral anticoagulants (the direct thrombin inhibitor dabigatran etexilate and the direct factor Xa inhibitors rivaroxaban and apixaban) have recently been approved for stroke prevention in atrial fibrillation.

**What's new**

Under the auspices of the Regional Anticoagulation Working Group, an expert panel convened to discuss practical, clinically important issues related to the long-term use of dabigatran etexilate for stroke prevention in non-valvular atrial fibrillation. This practical information will help clinicians make appropriate use of this new therapeutic option in daily clinical practice.

## Introduction

Atrial fibrillation (AF) is the most common sustained cardiac arrhythmia. It affects approximately 1–2% of the world population 1 and its prevalence increases with age 2. AF is associated with an increased risk of thromboembolism and is the most prevalent factor for cardioembolic stroke. Approximately, 15% of all strokes, and up to one-third of strokes affecting people over 80 years of age, occur in patients with AF 3.

Vitamin K antagonists (VKAs) have been the standard of care for stroke prevention in patients with AF since the early 1990s. They are very effective for the prevention of cardioembolic stroke (relative risk reduction of 64% vs. placebo) 4, but are limited by factors such as drug–drug interactions, food interactions, slow onset and offset of action, haemorrhage and need for routine anticoagulation monitoring to maintain a therapeutic international normalised ratio (INR) 5. These limitations have resulted in the underuse of VKAs 6. Even when they are prescribed, the level of anticoagulation with VKAs is frequently outside the therapeutic range, potentially compromising safety and efficacy 7.

Multiple new oral anticoagulants have been developed as potential replacements for VKAs for stroke prevention in AF 8–10. Most are small synthetic molecules that target thrombin (e.g. dabigatran etexilate) or factor Xa (e.g. rivaroxaban, apixaban, edoxaban, betrixaban, YM150). These drugs have predictable pharmacokinetics that allow fixed dosing without routine laboratory monitoring. The pharmacological properties of dabigatran etexilate are described in Table 1 11. Dabigatran etexilate is currently approved in many countries, including the United States, Canada, Japan and the European Union for stroke prevention in AF. Postmarketing studies are needed to evaluate the benefits and risks of new therapeutic agents in larger and more diverse populations than those included in randomised controlled trials, and in situations that represent real-world conditions. The Global Registry on Long-Term Oral Antithrombotic Treatment in Patients with Atrial Fibrillation (GLORIA-AF) aims to collect data on the safety and effectiveness of antithrombotic treatments, including VKAs and dabigatran etexilate, in over 50,000 patients with newly diagnosed non-valvular AF at significant risk for stroke [ClinicalTrials.gov identifiers: NCT01428765 (Phase 1) and NCT01468701 (Phase 2 and 3)].

**Table 1:** Dabigatran etexilate: pharmacological properties

Oral direct thrombin inhibitor
Double prodrug converted into its active metabolite dabigatran
Bioavailability: ∼ 6%
Time to peak plasma concentration (*C*max): 2 h
Half-life: single dose: 8–10 h; multiple dose: 12–17 h
Binds directly to thrombin with a high affinity and specificity (reversible inhibition)
Predictable anticoagulant effect (no need for coagulation monitoring)
Fixed dose
No interactions with food
Low risk of drug–drug interactions
Excreted unchanged via kidneys (85% renal elimination)

Under the auspices of the Regional Anticoagulation Working Group, a multidisciplinary group of experts in thrombosis and haemostasis from Central and Eastern Europe, an expert panel with expertise in AF convened to discuss practical, clinically important issues related to the long-term use of dabigatran for stroke prevention in non-valvular AF. Helpful guidance can also be found in two recent publications 12,13.

## Efficacy and safety of dabigatran etexilate compared with VKAs and other novel anticoagulants for stroke prevention in AF

### Dabigatran etexilate in the RE-LY study

Dabigatran etexilate was evaluated for stroke prevention in AF in the Randomised Evaluation of Long-Term Anticoagulation Therapy (RE-LY) study 14,15. RE-LY was a randomised non-inferiority trial designed to compare two fixed doses of dabigatran (110 mg twice daily and 150 mg twice daily), each administered in a blinded manner, with open-label use of warfarin adjusted locally to maintain an INR of 2.0–3.0 [Prospective, Randomised, Open, Blinded End-point (PROBE) study]. A total of 18,113 patients from 951 centres in 44 countries were enrolled. Mean age was 71 years and 63.6% of the patients were men. Half of the patients had received long-term therapy with VKAs. Mean CHADS_2_ [cardiac failure, hypertension, age, diabetes, stroke (doubled)] score was 2.1 (the proportion of patients with a CHADS_2_ score of 0–1, 2 or 3–6 was 31.9%, 35.6% and 32.5%, respectively). The median duration of the follow-up period was 2.0 years. In the warfarin group, mean time in therapeutic range (TTR) was 64%.

The main efficacy and safety results of the RE-LY study are described in Table 2. Both dabigatran doses were non-inferior to warfarin with respect to the primary efficacy outcome of stroke or systemic embolism. In addition, the 150-mg dose was superior to warfarin with respect to the primary efficacy outcome and significantly reduced both ischaemic and haemorrhagic stroke. The 110-mg dose significantly reduced haemorrhagic stroke only, with comparable efficacy to warfarin for ischaemic stroke. Myocardial infarction (MI) rates were similar with dabigatran and warfarin (see below).

**Table 2:** Main efficacy and safety results of the RE-LY study 14,15

	Dabigatran 110 mg (*N* = 6016)	Dabigatran 150 mg (*N* = 6076)	Warfarin (*N* = 6076)	Dabigatran 110 mg vs. Warfarin	Dabigatran 150 mg vs. Warfarin
**Efficacy outcomes**	(%/year)	(%/year)	(%/year)	RR (p-value*)	RR (p-value*)
Stroke or systemic embolism	1.54	1.11	1.71	0.90 (< 0.001†, 0.34)	0.66 (< 0.001†, < 0.001)
Ischaemic or unspecified stroke	1.34	0.92	1.21	1.11 (0.35)	0.76 (0.003)
Haemorrhagic stroke	0.12	0.10	0.38	0.31 (< 0.001)	0.26 (< 0.001)
Myocardial infarction	0.82	0.81	0.64	1.29 (< 0.09)	1.27 (< 0.12)
**Safety outcomes**	(%/year)	(%/year)	(%/year)	RR (p-value)	RR (p-value)
Major bleeding	2.87	3.32	3.57	0.80 (0.003)	0.93 (0.31)
Gastrointestinal bleeding	1.15	1.56	1.07	1.08 (< 0.52)	1.48 (< 0.001)
Intracranial bleeding	0.23	0.32	0.76	0.30 (< 0.001)	0.41 (< 0.001)

* p-value for superiority, except otherwise indicated;

† p-value for non-inferiority. RR, relative risk.

With respect to the primary safety outcome of major bleeding, both dabigatran doses were non-inferior to warfarin and the 110-mg dose was superior to warfarin. In addition, the rates of life-threatening bleeding and intracranial bleeding were significantly reduced with both doses of dabigatran. The rates of intracranial haemorrhage were 0.23%/year, 0.32%/year and 0.76%/year with the 110-mg dose, the 150-mg dose and warfarin, respectively (p < 0.001). Intracranial haemorrhage is the most devastating complication of VKA therapy and a major concern for clinicians; therefore, the relative risk reduction of 70% with the 110-mg dose and of 59% with the 150-mg dose represents an important advantage of dabigatran. A significant increase in the risk of gastrointestinal bleeding was observed with the 150-mg dose but not with the 110-mg dose. Dyspepsia occurred more frequently with both doses of dabigatran than with warfarin (see below).

Two recently published phase III trials have compared the oral direct factor Xa inhibitors rivaroxaban and apixaban, respectively, with warfarin for primary stroke prevention in patients with non-valvular AF.

### Rivaroxaban in the ROCKET AF study

The Rivaroxaban Once Daily Oral Direct Factor Xa Inhibition Compared with Vitamin K Antagonism for Prevention of Stroke and Embolism Trial in Atrial Fibrillation (ROCKET AF) study 16 was a double-blind, double-dummy trial comparing rivaroxaban 20 mg once daily (15 mg once daily in patients with a creatinine clearance between 30 and 49 ml/min) to dose-adjusted warfarin. A total of 14,264 patients from 1178 centres in 45 countries were randomised. Mean age was 73 years. Mean CHADS_2_ score was 3.47 (the proportion of patients with a CHADS_2_ score of 2 or 3–6 was 13% and 87%, respectively). In the warfarin group, mean TTR was 55%.

The main efficacy and safety results of the ROCKET AF study are described in Table 3. Rivaroxaban was non-inferior to warfarin with respect to the primary efficacy outcome (stroke or non-central nervous system systemic embolism) and the primary safety outcome (major bleeding). Efficacy was superior according to the on-treatment analysis but not according to the intention-to-treat analysis. MI rates were similar with rivaroxaban and warfarin. Rivaroxaban significantly reduced haemorrhagic stroke but not ischaemic stroke. The rates of intracerebral haemorrhage were 0.8%/year with rivaroxaban and 1.2%/year with warfarin, respectively (p < 0.02).

**Table 3:** Main efficacy and safety results (intention-to-treat analysis) of the ROCKET AF study 16

	Rivaroxaban (*N* = 7081)	Warfarin (*N* = 7090)	Rivaroxaban vs. Warfarin
**Efficacy outcomes**	(%/year)	(%/year)	HR (p-value*)
Stroke or systemic embolism	2.1	2.4	0.88 (< 0.001†, 0.12)
Ischaemic stroke	1.34	1.42	0.94 (0.58)
Haemorrhagic stroke	0.26	0.44	0.59 (0.024)
Myocardial infarction	0.91	1.12	0.81 (0.12)
**Safety outcomes**	(%/year)	(%/year)	RR (p-value)
Major bleeding	3.6	3.4	1.04 (0.58)
Gastrointestinal bleeding	3.15	2.16	(< 0.001)
Intracranial bleeding	0.5	0.7	0.67 (0.02)

* p-value for superiority, except otherwise indicated;

† p-value for non-inferiority. HR, hazard ratio; RR, relative risk.

Rivaroxaban is currently approved in many countries including the United States, Canada, Japan and the European Union for stroke prevention in AF.

### Apixaban in the ARISTOTLE study

The Apixaban for Reduction in Stroke and Other Thromboembolic Events in Atrial Fibrillation (ARISTOTLE) trial 17 was a double-blind, double-dummy trial comparing apixaban 5 mg twice daily (2.5 mg twice daily in two or more of the following criteria: age ≥ 80 years, body weight ≤ 60 kg or serum creatinine ≥ 1.5 mg/dl) with adjusted-dose warfarin. A total of 18,201 patients from 1034 centres in 39 countries were enrolled. Median age was 70 years and 57% of the patients had received long-term therapy with VKAs. Mean CHADS_2_ score was 2.1 (the proportion of patients with a CHADS_2_ score ≤ 1, 2 or ≥ 3 was 34%, 35.8% and 30.2%, respectively). The median duration of the follow-up period was 1.8 years. In the warfarin group, mean TTR was 62%.

The main efficacy and safety results of the ARISTOTLE study are described in Table 4. Apixaban was superior warfarin with respect to the primary efficacy outcome (stroke or systemic embolism) and the primary safety outcome (major bleeding according to ISTH criteria). In addition, the key efficacy outcome of all-cause mortality was reduced by 11% (p < 0.047). MI rates were similar with apixaban and warfarin. Apixaban significantly reduced haemorrhagic stroke but not ischaemic stroke. The rate of intracranial haemorrhage was 0.33%/year with apixaban and 0.8%/year with warfarin, respectively (p < 0.001).

**Table 4:** Main efficacy and safety results of the ARISTOTLE study 17

	Apixaban (*N* = 9120)	Warfarin (*N* = 9081)	Apixaban vs. Warfarin
**Efficacy outcomes**	(%/year)	(%/year)	HR (p-value*)
Stroke or systemic embolism	1.27	1.60	0.79 (< 0.01)
Ischaemic stroke or uncertain type of stroke	0.97	1.05	0.92 (0.42)
Haemorrhagic stroke	0.24	0.47	0.51 (< 0.001)
Myocardial infarction	0.53	0.61	0.88 (0.37)
**Safety outcomes**	(%/year)	(%/year)	RR (p-value)
Major bleeding (ISTH criteria)	2.13	3.09	0.69 (< 0.001)
Gastrointestinal bleeding	0.76	0.86	0.89 (0.37)
Intracranial bleeding	0.33	0.80	0.42 (< 0.001)

* p-value for superiority. HR, hazard ratio; ISTH, International Society on Thrombosis and Haemostasis.

Apixaban was recently approved in the European Union and in the United States for stroke prevention in AF.

### Indirect comparisons of dabigatran etexilate, rivaroxaban and apixaban for stroke prevention in atrial fibrillation

Several studies have indirectly compared dabigatran etexilate (150 mg twice daily and 110 mg twice daily), rivaroxaban and apixaban for efficacy and safety outcomes 18,19. Although no profound differences in efficacy and safety were reported, some differences were observed. For example, dabigatran etexilate 150 mg twice daily was superior to rivaroxaban for efficacy. Major bleeding was significantly lower with apixaban than with rivaroxaban or dabigatran etexilate 150 mg twice daily. In addition, major bleeding was significantly lower with dabigatran etexilate 110 mg twice daily than with rivaroxaban. However, such indirect intertrial comparisons should be used with caution and head-to-head studies will be required to confirm any differences in efficacy or safety between these drugs.

## Clinical use of dabigatran etexilate for stroke prevention in atrial fibrillation – practical considerations

### How to define ‘well-controlled’ VKA therapy? What are the benefits of dabigatran in ‘well-controlled’ VKA patients?

A patient who is ‘well controlled’ on VKA therapy is a patient whose INR fluctuates very little (i.e. is in the target range most of the time). The TTR, defined as the estimated total proportion of time that the INR is within the predetermined target range (INR 2.0–3.0) in an individual patient or in a given clinical setting, reflects the quality of anticoagulation. The most commonly used method of calculating the TTR is the Rosendaal method 20. The well-controlled patient on VKA therapy may be defined as a patient with an individual TTR over 70%, who handles VKA treatment and laboratory monitoring without problems 21.

In most studies, the TTR refers to clinical centres and reflects the proportion of patients that these centres are able to hold permanently in the therapeutic range. A strong association between centre-based TTR and the effectiveness and safety of VKA therapy has been observed across a large number of studies 22–25. However, in usual practice the level of INR control is frequently poor 26,27, and this should be kept in mind when generalising from the results of randomised clinical trials. In a systematic review of 67 studies which included over 50,000 patient receiving VKA therapy for a wide range of indications, van Walraven et al. found a mean TTR of 66.4% in randomised controlled trials, 65.6% in anticoagulation clinics and 56.7% in community practice 26. Another meta-analysis looking at patients receiving warfarin therapy for AF in the United States found a mean TTR of 63% in anticoagulation clinics vs. 51% in community practice 28. One of the clear benefits of novel oral anticoagulants is that they have predictable pharmacokinetics allowing fixed dosing without the need for routine laboratory monitoring.

**Figure 1:**
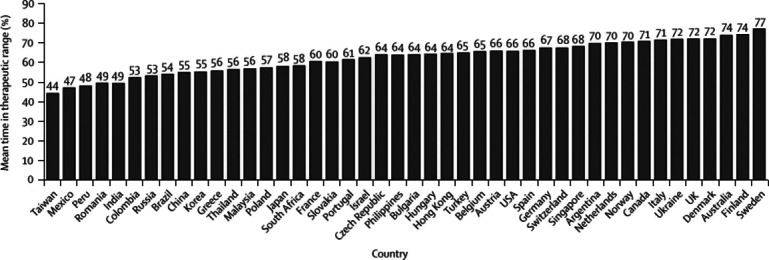
Country distribution of mean TTR in the RE-LY trial 29

A subgroup analysis of the RE-LY trial investigated the outcomes of the study in relation to each centre's mean TTR (cTTR) in warfarin-treated patients 29. The quartiles of cTTR for patients in the warfarin group were as follows: less than 57.1%, 57.1–65.5%, 65.5–72.6%, and greater than 72.6%. Mean cTTR ranged from 44% to 77% (country distribution of mean TTR is shown on Figure 1). There were no significant interactions between cTTR and prevention of stroke and systemic embolism with either dabigatran dose vs. warfarin. There was a significant interaction between cTTR and major bleeding when comparing the 150-mg dose of dabigatran with warfarin, with less bleeding events at lower cTTR but a similar event rate at higher cTTR, whereas rates of major bleeding were lower with the 110-mg dose of dabigatran than with warfarin irrespective of cTTR. The benefits of the 150-mg dose at reducing stroke and the 110-mg dose at reducing major bleeding vs. warfarin were consistent irrespective of centres' quality of INR control. The rates of intracranial bleeding were consistently lower in both dabigatran groups than in the warfarin group irrespective of cTTR. The benefits of dabigatran are primarily related with the fact that even compared with well-controlled warfarin therapy dabigatran reduces the risk of intracranial haemorrhage.

In VKA-treated patients, stability of the anticoagulant effect is often not achieved for several weeks after treatment initiation, and as a consequence the risks of stroke and bleeding are highest during this initial period 30. It is therefore important to include a significant proportion of VKA-naïve patients in trials comparing VKA therapy and new anticoagulants for stroke prevention in AF. The RE-LY trial was designed to enrol an equal proportion of VKA-naïve and VKA-experienced patients 31. Within assigned treatment groups, the only significant differences between VKA-naïve and VKA-experienced patients were observed in the dabigatran 110 mg group: cardiovascular death and the composite outcome of life-threatening bleeding, disabling stroke and death were less frequent in VKA-experienced patients. Previous VKA exposure did not influence the benefits of dabigatran etexilate at either dose compared with VKA therapy.

### Which patients are suitable for dabigatran? Which factors should be considered when selecting the dose for an individual patient?

Setting aside cost/reimbursement issues, most patients with AF requiring anticoagulant therapy would be suitable for dabigatran. Two compelling arguments to support the use of dabigatran in most patients are the superior efficacy of the 150-mg dose and the two-third reduction in intracerebral haemorrhage with both doses. An additional advantage of dabigatran over VKA therapy is that it does not require routine coagulation monitoring. Should financial constraints be considered, priority targets for dabigatran may be newly diagnosed patients, patients who are intolerant to VKA therapy, poorly controlled patients on VKA therapy, patients refusing VKA therapy and patients with previous stroke or developing thromboembolic events while on VKA therapy.

The dosage of dabigatran can be selected based on patient characteristics considering the superior efficacy of the 150-mg dose and the superior safety of the 110-mg dose. Clinicians are generally concerned about drug safety and may be tempted to use the lower dose. However, as a result of its superior efficacy, the higher dose should be strongly considered in the absence of specific risk factors for bleeding. Factors that may contribute to selecting the lower dabigatran dose include the thromboembolic risk, any risk of bleeding, drug interactions, age and moderate renal impairment (CrCl 30–50 ml/min). In patients with severe renal impairment (CrCl < 30 ml/min), treatment with dabigatran is contraindicated. Renal function should always be assessed prior to treatment initiation with dabigatran etexilate. During treatment, renal function should be assessed in clinical situations where it is suspected that it could decline or deteriorate. In patients with moderate renal impairment and in those over 75 years of age, renal function should be assessed at least once a year.

The RE-LY trial included 7258 patients aged ≥ 75 years and 3505 patients with moderate renal impairment (severe renal impairment was one of the exclusion criteria) 32. For the primary efficacy outcome of stroke or systemic embolism, there was no significant interaction between age or baseline renal function and dabigatran treatment. Rates of major bleeding increased with age and there was a significant interaction between age and treatment that attenuated the benefits of dabigatran with increasing age. For patients aged < 75 years, both doses of dabigatran reduced the risk of major bleeding compared with warfarin. In patients aged ≥ 75 years, the rate of major bleeding was similar with dabigatran 110 mg twice daily compared with warfarin, whereas a trend towards a higher risk of major bleeding was observed with dabigatran 150 mg twice daily 33. An interaction between renal function and treatment was no longer evident after adjustment for age. There was no interaction between either age or renal function and the benefits of dabigatran vs. warfarin in reducing haemorrhagic stroke. The benefit of dabigatran vs. warfarin for stroke prevention is independent of age and renal function. The benefit of dabigatran vs. warfarin in reducing extracranial bleeding is significantly attenuated with increasing age, whereas the reduction in haemorrhagic stroke is maintained in older patients.

Patients aged ≥ 80 years and patients treated concomitantly with dabigatran and verapamil should be treated with the lower dose of dabigatran. In patients aged between 75 and 80 years, the highest dose should be used; however, the lower dose can be individually considered, at the discretion of the physician, when the thromboembolic risk is low and the bleeding risk is high. For patients with gastritis, oesophagitis or gastroesophageal reflux, the lower dose may be considered. In patients with moderate renal impairment (CrCl 30–50 ml/min), the recommended dose is 150 mg twice daily; however, the lower dose should be considered in patients with a high risk of bleeding 13.

### What are the main clinically relevant interactions between dabigatran etexilate and other drugs?

Dabigatran has a low potential for interactions with other drugs. It does not interact with the cytochrome P450 system. As the prodrug dabigatran etexilate is a substrate of the P-glycoprotein (P-gp) efflux transporter, the main clinically relevant interactions are with drugs that are strong inhibitors or inducers of P-gp 13,21. The main clinically relevant drug interactions are listed in Table 5. Concomitant administration of atorvastatin, diclofenac, pantoprazole, clopidogrel and digoxin is possible without dose adjustment 13.

**Table 5:** Main clinically relevant interactions between dabigatran etexilate and other drugs 13

P-glycoprotein inhibitors	Ketoconazole (systemic) Cyclosporine Itraconazole Tacrolimus Dronedarone	Concomitant administration is contraindicated
	Posaconazole	Concomitant administration is not recommended
	Amiodarone Quinidine	Concomitant administration requires caution and bleeding risk assessment
	Verapamil	Concomitant administration requires caution, bleeding risk assessment and dabigatran dose adjustment (110 mg twice daily)
P-glycoprotein inducers	Rifampicin St. John's Wort (*Hypericum perforatum*) Carbamazepine Phenytoin	Concomitant administration should be avoided
Other drugs interacting with P-glycoprotein	Protease inhibitors (e.g. ritonavir, tipranavir, nelfinavir, saquinavir)	Concomitant administration is not recommended

Caution is recommended when considering concomitant prescription of dabigatran etexilate and non-steroidal anti-inflammatory drugs, antiplatelet agents, selective serotonin reuptake inhibitors or serotonin–norepinephrine reuptake inhibitors. Co-administration of a proton pump inhibitor is unlikely to reduce the anticoagulant effect of dabigatran etexilate.

### How would you manage a dabigatran-treated patient with acute coronary syndrome? How to manage an AF patient if coronary intervention with stent implantation is necessary?

Acute coronary syndromes occurring in patients on dabigatran therapy should be diagnosed and treated with early angiography and intervention. Dabigatran is generally discontinued temporarily during the acute phase, although an uninterrupted anticoagulation strategy may be preferred in moderate- to high-risk patients 34.

Following percutaneous coronary intervention (PCI) with stenting, dual antiplatelet therapy (aspirin and clopidogrel) is mandatory, and in patients with AF triple therapy combining dual antiplatelet therapy and oral anticoagulation should be used 34.

Concomitant administration of a VKA and one or two antiplatelet agents is associated with a substantial increased risk of major bleeding events compared with monotherapy 35–37. However, oral anticoagulant therapy improves prognosis (reduced mortality and major adverse cardiac events) despite the increase in major bleeding, even in patients with a high bleeding risk 38. In patients treated with warfarin or either dose of dabigatran in the RE-LY trial, concomitant treatment with aspirin, clopidogrel or both was associated with increased major bleeding rates 33. Among, approximately, 1000 patients who received concomitant treatment with both aspirin and clopidogrel, the rates of major bleeding were 4.72%, 4.66% and 5.21% in the groups receiving dabigatran 110 mg twice daily, dabigatran 150 mg twice daily and warfarin, respectively 33. During the period when triple therapy is used, the lower dose of dabigatran (110 mg twice daily) would appear to be appropriate, in line with the recommendation by recent European and North American consensus documents to maintain an INR at the lower end of the therapeutic range (2.0–2.5) in VKA-treated patients 39,40. Both consensus documents emphasise that limiting the duration of triple therapy when possible is a key step to reduce the bleeding risk. However, North American experts recommend a much longer duration of triple therapy than European experts as they place greater emphasis on reducing the risk of thrombosis. Both documents recommend the use of bare metal stents rather than drug eluting stent in patients with increased bleeding risk 41. Further studies are needed to evaluate the efficacy and safety of dabigatran in combination with dual antiplatelet therapy.

### Was there a difference in the rate of MI with dabigatran compared with VKAs in the RE-LY study?

In the original report of the RE-LY study, the rate of MI was significantly higher with both doses of dabigatran than with warfarin 14. However, a subsequent analysis performed following discussion with the FDA, which included additional data on silent MIs (based on the new appearance of pathologic Q waves on ECG), did not reveal significant differences between dabigatran and warfarin 15. A detailed post hoc analysis of the RE-LY study aimed to provide a better understanding of the effects of dabigatran on myocardial ischaemic events was recently published 42. This analysis reported rates of MI and other clinical events related to myocardial ischaemia: unstable angina, cardiac death, cardiac arrest, coronary artery bypass graft (CABG) surgery and PCI. A non-significantly higher number of MIs was observed with both doses of dabigatran compared with warfarin; there was no excess of other myocardial ischaemic events. The composite of stroke, systemic embolism, MI, unstable angina, cardiac death, cardiac arrest, CABG, PCI and major bleeding occurred less frequently with dabigatran than with warfarin (the difference was statistically significant for the 150-mg dose).

A meta-analysis evaluated the risk of MI or acute coronary syndrome (ACS) in seven randomised controlled trials comparing dabigatran with warfarin, enoxaparin or placebo for stroke prevention in AF, VTE prevention in major orthopaedic surgery, VTE treatment and prevention of cardiovascular events in patients with ACS 43. The RE-LY study accounted for 59% of the patients and 74% of the events included in the meta-analysis. The risk of MI or ACS was found to be increased with dabigatran compared with the various control treatments. Although the relative risk increase was 33%, the absolute risk increase was very small (0.27%). In the six studies reporting on mortality, overall mortality was significantly lower with dabigatran compared with control treatments.

Warfarin has been shown to be very effective in preventing reinfarction in patients with previous MI 44,45. Therefore, the most plausible explanation for the relative increase in acute coronary events with dabigatran compared with warfarin is not that dabigatran causes coronary events but rather that warfarin may provide a greater coronary protective effect in high-risk patients 46,47.

The 2012 focused update of the European Society of Cardiology for the management of AF state that in a dabigatran-treated patient presenting with an acute coronary syndrome, the concerned clinician may consider the use of a VKA or a factor Xa inhibitor, although there is little evidence to support this approach 48. In the RE-LY study, 5650 patients had a history of coronary artery disease (CAD) and/or previous MI. The effects of dabigatran etexilate compared with warfarin were highly consistent between patients with and without prior CAD and/or MI 42.

### How to manage a dabigatran-treated patient who presents with an ischaemic stroke?

A patient treated with dabigatran etexilate may develop an acute ischaemic stroke. Clinical experience with the use of thrombolytic therapy in patients treated with dabigatran is very limited: only isolated case reports have been published 49–54. If the patient's activated partial thromboplastin time (aPTT) is prolonged, it should be assumed that the patient is anticoagulated and intravenous administration of recombinant tissue plasminogen activator should not be performed 48. The thrombin clotting time (TT) and the ecarin clotting time (ECT), but not the INR, are also appropriate tests to assess coagulation status in dabigatran-treated patients who are considered possible candidates for thrombolysis (see below) 13.

Until more evidence is available, it appears reasonable to resume treatment with dabigatran etexilate immediately after a transient ischaemic attack, 3–5 days after a minor or moderately severe stroke, and 10–14 days after a severe stroke 55.

### How to use dabigatran in patients undergoing electrical or pharmacological cardioversion?

Cardioversion (both electric and pharmacological) in patients with AF is associated with an increased risk of thromboembolic events. In the RE-LY study, cardioversion on randomised treatment was permitted. The study protocol recommended maintenance of the assigned study drug during cardioversion. As a safety measure, transoesophageal echocardiography (TEE) was encouraged. All patients who underwent cardioversion during their participation in the RE-LY trial were included in a subgroup analysis 56. A total of 1983 cardioversions were performed in 1270 patients during the course of the trial. TEE was used only in a minority of procedures (165/647 in the dabigatran 110-mg group, 162/672 in the dabigatran 150-mg group and 88/664 in the warfarin group). The rates of stroke and major bleeding within 30 days of cardioversion on both doses of dabigatran were low and comparable to those on warfarin with or without TEE. Based on the available data, it appears safe to continue treatment with dabigatran etexilate in patients undergoing cardioversion.

### How should twice-daily dosing be considered in relation to the benefits in efficacy and safety seen with dabigatran etexilate?

The twice-daily dosing of dabigatran may be one of the reasons why dabigatran has been demonstrated to improve efficacy and safety compared with warfarin. Pharmacokinetic simulations show that a twice-daily regimen results in less daily fluctuations in plasma concentrations of dabigatran, thereby minimising the risks of both thrombosis and bleeding 57. On the other hand, twice-daily dosing may be a disadvantage with regard to treatment adherence. A systematic review evaluating the effect of medication dosing frequency on adherence in chronic diseases found that patients receiving once-daily dosing had 2–44% more adherent days compared with patients receiving twice-daily dosing, with most studies clustering around 13–26% 58. Long-term rates of compliance with prophylactic therapies in patients who have no symptoms are usually problematic, and therefore clinicians need to carefully monitor the compliance of patients treated with dabigatran. A conceptual model of adherence to oral anticoagulants in patients with AF was recently developed based on a literature review and patient focus groups 59. This model identifies an adherence process that may guide interventions, such as educational and behavioural programmes, aimed at improving adherence to anticoagulation therapy.

### How to manage dabigatran-treated patients who develop dyspepsia?

In the RE-LY study, there was a significantly increased risk of developing dyspepsia with both doses of dabigatran compared with warfarin (11.8% with dabigatran 150 mg twice daily, 11.3% with dabigatran 110 mg twice daily and 5.8% with warfarin; p < 0.001) 14. This adverse event may be linked to the tartaric acid core of dabigatran capsules (low pH enhances the absorption of the drug). Although there was a statistically significant increase in the rate of gastrointestinal bleeding with the 150-mg dose of dabigatran compared with warfarin (relative risk, 1.48; p < 0.001) – no difference was observed with the 110-mg dose of dabigatran (relative risk, 1.08; p = 0.52) – there is no established link between dyspepsia and the risk of gastrointestinal bleeding.

Based on limited clinical experience, it may be beneficial to take dabigatran with meals or a large glass of water. Sucralfate or drugs that increase gastric pH (antacids, proton pump inhibitors) may be helpful. If dyspepsia is significant and cannot be explained by other reasons, the patient may be switched to an alternative treatment.

### How to manage major bleeding in patients treated with dabigatran? How to antagonise the anticoagulant effect of dabigatran?

The management of bleeding in patients on dabigatran therapy should be focused on treatment discontinuation and supportive measures. There is currently no specific antidote to dabigatran 60; however, an antidote is currently being developed. The source of bleeding should be identified and management should be tailored according the severity and location of the haemorrhage. Importantly, diuresis should be maintained, since dabigatran is excreted via the kidneys. Plasma levels of dabigatran decrease relatively quickly in patients with normal renal function because of its relatively short half-life, and the bleeding risk 12 h after the last dose should be acceptably low. Meanwhile, supportive measures to control the bleeding should be taken. In case of severe or life-threatening haemorrhage, haemodialysis may be considered, dabigatran being dialysable because of its relatively low plasma protein binding 60. The use of reversal agents such as recombinant activated factor VII or prothrombin complex concentrates may be an option, although their utility and benefit-risk ratio have not been established 60,61.

### How to proceed in a dabigatran-treated AF patient who requires an invasive or surgical procedure?

In patients who require elective surgery, dabigatran should be discontinued. The timing of discontinuation will depend on the complexity of the surgery, the risk of bleeding and the patient's renal function (Table 6).

**Table 6:** Dabigatran etexilate discontinuation rules before invasive or surgical procedures

Renal function (CrCl, ml/min)	Estimated half-life (h)	Timing of dabigatran discontinuation	

		High risk of bleeding or major surgery	Standard risk
≥ 80	∼ 13	2 days before	24 h before
50–80	∼ 15	2–3 days before	1–2 days before
30–50	∼ 18	4 days before	2–3 days before

If renal function is normal, plasma levels will decrease to approximately 25% of steady-state levels 24 h after discontinuation, approximately 12–15% after 36 h and approximately 5–10% after 48 h. Patients with moderate renal impairment will require a longer period of discontinuation prior to surgery. In these patients, it is important to assess the coagulation status prior to surgery.

If an acute intervention is required, dabigatran should be temporarily discontinued. If possible, the invasive or surgical procedure should be delayed until at least 12 h after the last dose. If the intervention cannot be delayed, the risk of bleeding should be weighed against the urgency of the procedure.

In contrast to patients on VKA therapy, dabigatran-treated patients do not require bridging therapy with low-molecular-weight heparin because of the rapid onset and offset of action of dabigatran etexilate 48.

Experience with dabigatran etexilate for periprocedural anticoagulation in patients undergoing AF ablation is limited 62–64. Lakkireddy et al. reported that dabigatran use (with treatment interruption on the morning of the procedure) was associated with more thromboembolic and bleeding complications compared with uninterrupted warfarin therapy 62. Using a similar protocol in patients treated with dabigatran 110 mg twice daily, Kaseno et al. observed no symptomatic thromboembolic complications and less bleeding complications than with warfarin 63. Winkle et al. discontinued dabigatran between 36 and 60 h before the procedure depending on estimated glomerular filtration rate and observed no bleeding and thromboembolic complications until 30 days after the procedure 64. Recent guidelines recommend performing catheter ablation on uninterrupted anticoagulation in AF patients on VKA therapy 48,65. Studies are required to evaluate the efficacy and safety of this approach in dabigatran-treated patients.

### What can be recommended with regard to laboratory monitoring for dabigatran in exceptional situations?

Dabigatran has a predictable pharmacokinetic profile which avoids the need for routine coagulation monitoring. However, in specific clinical circumstances (e.g. suspected overdose, dabigatran-treated patients presenting in emergency departments), it may be advisable to assess the anticoagulant status of the patient 60,66.

The TT assay directly assesses the activity of thrombin in a plasma sample and allows measurement of the activity of the direct thrombin inhibitors. TT tests are available in many hospital laboratories.

TT is well correlated with dabigatran concentrations. The actual TT test measure will depend on the coagulometer and on the thrombin lot used for the measurement. It is therefore advisable to use the calibrated Hemoclot® Thrombin Inhibitor assay (Hyphen BioMed, Neuville-sur-Oise, France) with direct calibration with stable, lyophilised dabigatran standards to calculate the dabigatran concentration rather than to determine TT only.

The aPTT targets the intrinsic pathway of the coagulation cascade. Prolongation of the aPTT occurs when dabigatran plasma concentrations increase, but the aPTT concentration response curve is curvilinear and flattens at higher concentrations. The aPTT may be useful in determining an excess of anticoagulant activity.

Prothrombin time (PT) and the INR represent the clotting time in the extrinsic coagulation pathway. Dabigatran has little effect on PT and INR at clinically relevant plasma concentrations. Therefore, INR tests should not be performed.

The activated clotting time (ACT) is a quantitative assay based on a similar test principle to aPTT. It is frequently used as a bedside assay to measure the anticoagulant effect of unfractionated heparin in patients undergoing PCI or CABG surgery. There are limited data for ACT with dabigatran.

The ECT is a specific assay for thrombin generation; it provides a direct measure of the activity of direct thrombin inhibitors. There is a close linear correlation between ECT prolongation and plasma concentrations of dabigatran. So far the ECT has been used as a research tool with limited access. The development of commercial kits may improve the availability and practicality of this test.

In summary, PT is not an appropriate test to assess the anticoagulant effect of dabigatran; aPTT is helpful to detect the presence of dabigatran but not to evaluate its concentration; the Hemoclot® Thrombin Inhibitor test and the ECT are sensitive tests for quantitating the anticoagulant effect of dabigatran.

## Conclusion

Vitamin K antagonists have been shown to be highly effective for stroke prevention in patients with AF. However, their well-known limitations, which include interactions with numerous foods and drugs as well as the need for frequent coagulation monitoring and dose adjustments, have resulted in both underuse and suboptimal use. The development of new oral anticoagulant agents that have been shown to be at least as effective as VKAs with a lower bleeding risk, no food interactions, few drug interactions and no requirement for routine laboratory monitoring, open a new era in the prevention of thromboembolic events in patients with AF. Dabigatran etexilate, the first of these new oral anticoagulants to be approved by the United States Food and Drug Administration and the European Medicines Agency in this indication, represents an effective and safe alternative to VKAs. The practical information reviewed in this article will help clinicians make appropriate use of this new therapeutic option in daily clinical practice.
